# Reliable, rapid, and remote measurement of metacognitive bias

**DOI:** 10.1038/s41598-024-64900-0

**Published:** 2024-06-28

**Authors:** Celine A. Fox, Abbie McDonogh, Kelly R. Donegan, Vanessa Teckentrup, Robert J. Crossen, Anna K. Hanlon, Eoghan Gallagher, Marion Rouault, Claire M. Gillan

**Affiliations:** 1 Department of Psychology, Trinity College Dublin, Dublin, Ireland; 2 Trinity College Institute for Neuroscience, Trinity College Dublin, Dublin, Ireland; 3 Paris Brain Institute (ICM), Centre National de la Recherche Scientifique (CNRS), Paris, France; 4 ADAPT Centre for Digital Technology, Trinity College Dublin, Dublin, Ireland

**Keywords:** Human behaviour, Perception, Decision

## Abstract

Metacognitive biases have been repeatedly associated with transdiagnostic psychiatric dimensions of ‘anxious-depression’ and ‘compulsivity and intrusive thought’, cross-sectionally. To progress our understanding of the underlying neurocognitive mechanisms, new methods are required to measure metacognition remotely, within individuals over time. We developed a gamified smartphone task designed to measure visuo-perceptual metacognitive (confidence) bias and investigated its psychometric properties across two studies (N = 3410 unpaid citizen scientists, N = 52 paid participants). We assessed convergent validity, split-half and test–retest reliability, and identified the minimum number of trials required to capture its clinical correlates. Convergent validity of metacognitive bias was moderate (r(50) = 0.64, *p* < 0.001) and it demonstrated excellent split-half reliability (r(50) = 0.91, *p* < 0.001). Anxious-depression was associated with decreased confidence (β =  − 0.23, SE = 0.02, *p* < 0.001), while compulsivity and intrusive thought was associated with greater confidence (β = 0.07, SE = 0.02, *p* < 0.001). The associations between metacognitive biases and transdiagnostic psychiatry dimensions are evident in as few as 40 trials. Metacognitive biases in decision-making are stable within and across sessions, exhibiting very high test–retest reliability for the 100-trial (ICC = 0.86, N = 110) and 40-trial (ICC = 0.86, N = 120) versions of Meta Mind. Hybrid ‘self-report cognition’ tasks may be one way to bridge the recently discussed reliability gap in computational psychiatry.

## Introduction

Metacognition, the ability to reflect upon and evaluate cognitive experiences, is a central facet of human consciousness^1^. As feedback is often absent in daily life, metacognition facilitates the continuous monitoring of our moment-to-moment decisions, informing—for better or worse—our beliefs about our skills, abilities^2^ and even self-worth^3^. Aside from informing self-concepts, at a more granular level, metacognition facilitates learning and guides behaviours in the absence of direct feedback^4,5^, and allows us to communicate uncertainty in decision-making to others^6^. The prototypical example of metacognition is the confidence we hold in our own decisions^7^. By gathering repeated confidence judgements from individuals as they make choices, we can measure important facets of metacognition: bias and efficiency^8^. Bias refers to the tendency to give high or low confidence ratings on average, while efficiency is the extent to which our confidence levels reliably discern correct from incorrect decisions^9^.

Crucially, individuals vary in their metacognitive abilities and these differences correspond to where an individual sits along a spectrum of transdiagnostic mental health symptoms^10,11^. Specifically, a transdiagnostic dimension of ‘anxious-depression’ is linked to underconfidence in one’s own performance, while a separate dimension ‘compulsivity and intrusive thought’ is related to elevated confidence^12–16^. Metacognitive bias in these studies was originally calculated from confidence judgements at the ‘local’ trial-level, but recent work has shown that metacognition manifests across a hierarchy^3^. Within this hierarchy, isolated local confidence evaluations in single decisions are aggregated slowly over time to form more ‘global’ beliefs about one’s ability in a given domain, and may generalise into broader self-beliefs^2^. Indeed, recent work has shown that disturbances in local confidence across transdiagnostic psychiatric dimensions are reflected in similar patterns of biased global self-performance evaluations spanning longer timescales^16^. If generalised outside of a single domain, these biases could conceivably contribute over time to generalised negative schemata central to cognitive models of depression^17^.

To date, investigations have been largely cross-sectional and between-person^12,14–16^. This limits what we can learn about temporal dynamics, or cause and effect. For example, while it is possible that metacognitive biases in depression at the local level play a role in shaping global self-beliefs, it is equally plausible that changes in self-esteem reciprocally impact local confidence^3^. To address this gap, recent work has begun to adopt within-person designs, measuring metacognition within the same person over time. These studies have provided evidence to suggest that metacognitive biases fluctuate over time in healthy individuals^18^ and negative confidence bias reduces following cognitive behavioural therapy and antidepressant medication^13^. This suggests metacognitive bias is not a fixed or final trait, but instead may be malleable, and potentially a target for intervention. However, it remains poorly understood how these biases temporally relate to changes in psychopathology. One way to sample metacognition densely over time, and spanning periods of significant clinical change within an individual, is through online, remote data collection.

Prior studies have achieved remote testing by using web-based metacognitive tasks and recruiting well-powered samples through crowd-sourcing platforms, such as Amazon Mechanical Turk and Prolific^12,14–16^. However, paid crowd-sourced samples have come under scrutiny for generating poor quality data^19^, which is not necessarily resolved with the established protective quality measures^20^. As an alternative recruitment avenue, ‘Citizen Science’ is a valuable paradigm with improved data quality^21^, in which individuals participate in research voluntarily, due to motivational factors unrelated to financial gain^22^. Uncompensated, self-selected online samples provide comparable data quality to lab-based perceptual experiments, with the advantage of speeding up and scaling up data collection^23^, and being more representative^22^. Employing cognitive tasks through smartphone applications specifically has proven particularly beneficial in ensuring high-quality data collection among citizen scientists^24–27^.

One barrier to this, however, is growing concerns that many of the most commonly used cognitive tests in psychiatry suffer from poor reliability^28,29^. This is in contrast to self-report clinical questionnaires, which typically demonstrate good to excellent reliability^30,31^. Metacognitive bias differs from standard objective task outcomes, as it is typically measured using a hybrid approach that incorporates elements of cognitive assessment and self-report. Much like a typical cognitive test (and unlike a self-report questionnaire), some metacognitive tasks tightly control actual “Type I” performance (e.g., by titrating the task difficulty to each person’s ability), thereby preventing actual performance differences from confounding metacognitive judgements^32^. A key metric of metacognitive abilities, however, is not behavioural, but subjective—the estimate of confidence in one’s decisions^9^. For these reasons, metacognitive bias might enjoy a higher level of test–retest reliability that is more similar to a questionnaire than classic cognitive tests. Poor reliability is an issue for developments in the field of computational psychiatry, as prior findings on inter-individual differences may be imprecise and invalid^33^. Alternatively, a less pessimistic view of poor reliability among cognitive outcomes is that behavioural tasks provide legitimate estimates of momentary cognitive capacities when tested, but these are liable to fluctuations over time^34^. In line with this, cognitive capacities tend to temporally covary with affect and practice factors, which are often not accounted for in reliability assessments^35^. This further illustrates the need for within-subject longitudinal assessments in computational psychiatry, to infer individual clinical and cognitive phenotypes.

The present study aimed to test this using a brief gamified smartphone task called ‘Meta Mind’, designed to measure metacognitive bias reliably, remotely, and in as few trials as possible. To this end, we evaluated the psychometric properties of Meta Mind, including reliability (split-half and test–retest), and convergent validity, across two experiments. In the first experiment, paid participants completed Meta Mind and a traditional perceptual-decision making task, from which Meta Mind was adapted^13^. In the second experiment, a large sample of over 3000 citizen scientists completed Meta Mind and mental health questionnaires within the smartphone app. Visuo-perceptual decision-making tasks have become a staple method for measuring confidence^36^, providing estimates of domain general metacognition^15^, and so this was the task type upon which Meta Mind was based^13^. However, prior work has shown that this type of task is criticised by participants and described as tedious, lengthy and difficult^37^. This is a threat to research quality, as a lack of task engagement can increase rates of careless or inattentive responding, in some cases leading to spurious associations between cognition and self-reported psychopathology^38^. To address this, we focused on not just gamification, but determining the minimum number of trials required to measure metacognitive bias, while retaining adequate reliability and well-established clinical correlates.

## Results

### Experiment 1

#### Comparing Meta Mind and the traditional task

**Figure 1:**
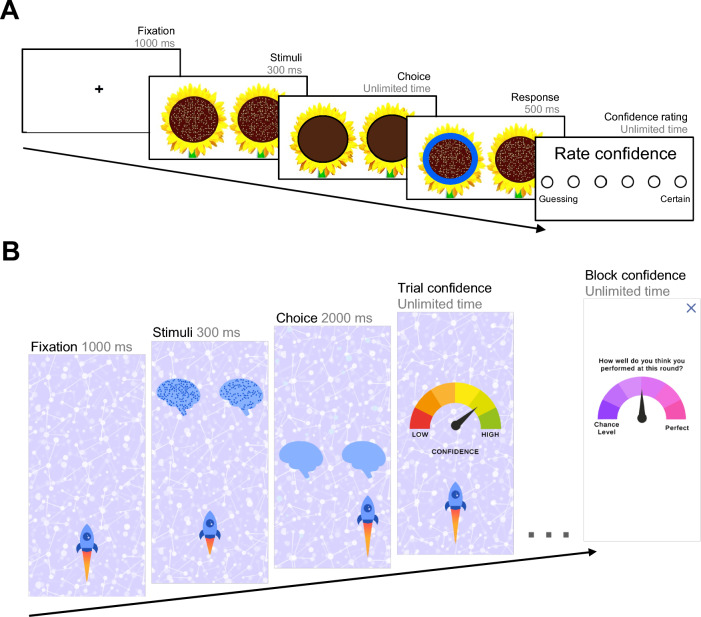
Task designs. (**A**) Perceptual decision-making task design (N = 210 trials). On each trial, participants are shown a fixation cross for 1000 ms (ms) before being shown two sunflowers with a different number of seeds for 300 ms. Participants are asked to judge and choose the sunflower contained more seeds (i.e., higher number of dots). This chosen sunflower is highlighted blue for 500 ms. Participants then have unlimited time to provide a confidence rating on their decision. (**B**) In Meta Mind, players are instructed to navigate their ship to the stimuli with more dots. At the start of each Meta Mind round (N = 20 trials), players are shown a screen with no icons for 1000 ms. Icons then appear at the top of the screen and drift towards the bottom. Icons contain dots for 300 ms. After the dots disappear, blank icons appear on screen for up to 2000 ms, until a choice is made. Players tap the left or right side of the screen to choose the correct stimuli. Missed trials are recorded and repeated with icons that have the same dot difference. After Meta Mind players choose a stimuli, they then rate their local confidence in the accuracy of that choice on the trial, with unlimited time. After 20 Meta Mind trials, players evaluate their overall accuracy on that block (round-level, global confidence).

**Figure 2:**
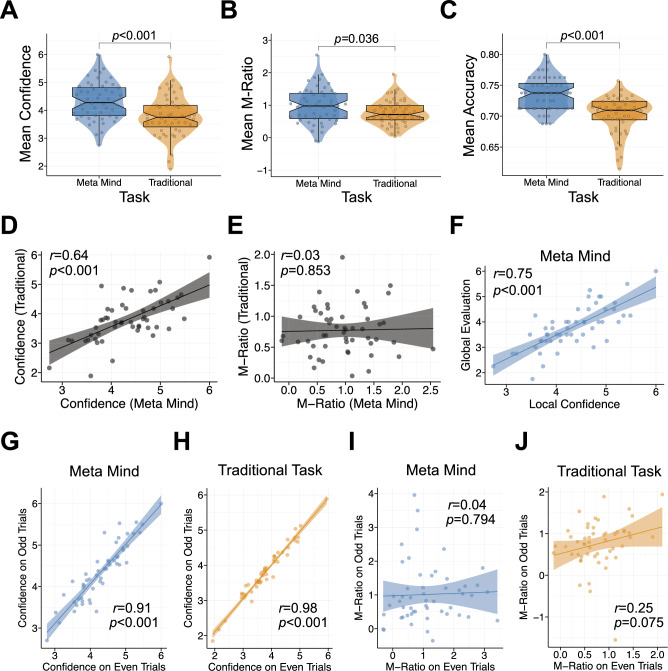
Comparing Meta Mind against the traditional metacognitive task (N = 52). Mean confidence (**A**), M-Ratio (**B**) and mean accuracy (**C**) were all significantly higher with Meta Mind (blue), compared to the traditional task (orange). While mean local confidence was significantly associated across tasks (**D**), there was no association between M-Ratio measure through Meta Mind and the traditional task (**E**). In Meta Mind, those with higher mean local confidence had higher mean global evaluations of performance (**F**). There was a high correlation between odd and even trials for mean confidence on both metacognitive tasks, indicating sufficient split-half reliability (**G**, **H**). Split-half reliability was poor for M-Ratio across tasks (**I**, **J**).

A sunflower-themed visuo-perceptual decision-making task was used as the traditional metacognition task (Fig. 1A)^13^, from which Meta Mind was designed (Fig. 1B). Comparing the tasks, Meta Mind was relatively shorter, taking on average 7.86 min (SD = 1.86) to complete, while the traditional task took 21.50 min (SD = 6.87) (β = 1.60, SE = 0.12, t = 13.15, *p* < 0.001). Figure 2 shows performance characteristics across the two metacognitive tasks. Metacognitive bias in Meta Mind, operationalised as mean local confidence, was significantly higher (M = 4.33, SD = 0.67) than in the traditional task (M = 3.80, SD = 0.74) (β = -0.70, SE = 0.18, t = -3.81, *p* < 0.001) (Fig. 2A). In addition to metacognitive bias, we also quantified metacognitive efficiency via M-Ratio (i.e., the ratio of metacognitive sensitivity to mean accuracy, where sensitivity is the extent to which confidence ratings discriminate between correct and incorrect trials^39^). M-Ratio was higher for Meta Mind (M = 0.97, SD = 0.54) compared to the traditional task (M = 0.77, SD = 0.38) (β =  − 0.41, SE = 0.19, t =  − 2.13, *p* = 0.036) (Fig. 2B). Despite the use of a staircase procedure, task accuracy was higher in Meta Mind (M = 74%, SD = 3) than the traditional task (M = 70%, SD = 3) (β =  − 1.03, SE = 0.17, t =  − 6.08, *p* < 0.001) (Fig. 2C). As expected, the traditional task had a significantly higher mean dot difference (M = 41.89, SD = 15.38) than Meta Mind (M = 7.91, SD = 2.81) (β = 1.83, SE = 0.08, t = 23.31, *p* < 0.001), given the constraints with increasing the dot number on relatively smaller phone screens when designing Meta Mind. There was modest evidence for learning effects for task difficulty only. Those who completed Meta Mind after first completing the traditional task achieved a higher level of objective difficulty (i.e., a lower dot difference) on the Meta Mind game (M = 8.92, SD = 3.13 vs. M = 6.91, SD = 2.05, β =  − 0.36, SE = 0.13, t =  − 2.74, *p* = 0.008). The analogous effect was not significant for the traditional task (β =  − 0.18, SE = 0.14, t =  − 1.29, *p* = 0.202). There were no effects of task order on mean confidence, M-Ratio or accuracy in either task (all *p* > 0.19).

Mean local confidence was moderately correlated across tasks (*r*(50) = 0.64, *p* < 0.001), indicating adequate convergent validity (Fig. 2D). Within Meta Mind, local confidence and global self-performance evaluations were correlated (*r*(50) = 0.75, *p* < 0.001) (Fig. 2F). The split-half reliability of local mean confidence was excellent for both Meta Mind (*r*(50) = 0.91, *p* < 0.001) and the traditional task (*r*(50) = 0.98, *p* < 0.001) (Fig. 2G,H). In contrast, there was no significant association between M-Ratio across task versions (*r*(50) = 0.03, *p* = 0.853) (Fig. 2E) and split-half reliability for M-Ratio in Meta Mind neared 0 (*r*(50) = 0.04, *p* = 0.794) and was poor for the traditional task (*r*(50) = 0.25, *p* = 0.075) (Fig. 2I,J). This follows recent work demonstrating that 100 should be considered the lowest boundary for the sufficient number of trials when estimating M-Ratio^39^, as Meta Mind and the traditional task only had 40 and 105 trials in each split (odd vs. even), respectively.

### Experiment 2

#### Test–retest reliability with the 100-trial version of *Meta* Mind

**Figure 3:**
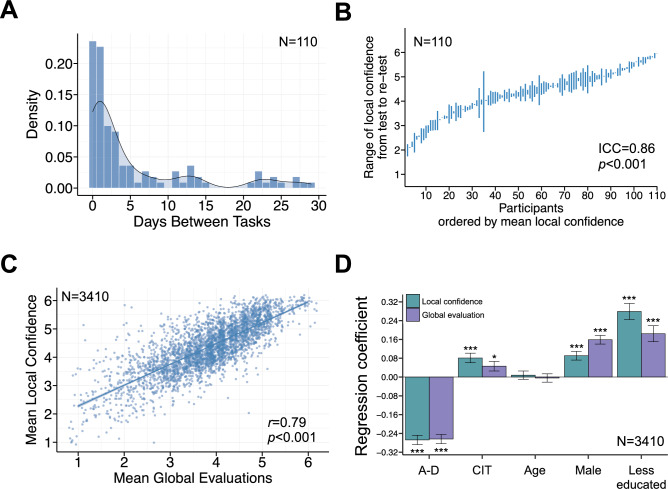
Validation of Meta Mind in a large sample of unpaid citizen scientists. N = sample size, ICC = intra-class correlation coefficient, *r* = correlation coefficient, *p* = *p* value, A-D = anxious-depression, CIT = compulsivity and intrusive thought, *** = *p* < 0.001, ** = *p* < 0.01, * = *p* < 0.05. (**A**) Density plots of days between Meta Mind test and retest completion (median = 2, SD = 7.74) (N = 110). (**B**) There was good test–retest reliability for mean confidence among sub-samples of citizen scientists that played 100 trials of Meta Mind twice within 30 days (N = 110). (**C**) Those with higher local, trial-level confidence had elevated, round-level global evaluations of task performance (N = 3410). (**D**) Among N = 3410, mean local confidence and global evaluations were higher among males and those that were less educated. Those with higher levels of anxious-depression **(**A–D) had lower local confidence and global evaluations, while those with higher levels of compulsivity and intrusive thought (CIT) have elevated local confidence. The positive association between CIT and mean global evaluations was marginally significant at *p* = 0.023. The error bars represent the standard error around the standardised beta coefficient.

Among the 110 unpaid, citizen scientists that played the 100-trial version of Meta Mind twice, the median time interval between test and retest game completion was 2 days (SD = 7.74) (Fig. 3A). An intra-class correlation (ICC; two-way mixed-effects model with absolute agreement, single rater) of 0.86, with 95% confident interval = 0.80–0.90, *p* < 0.001), was calculated for local confidence bias among repeated Meta Mind players, indicating good test–retest reliability^40^ (Fig. 3B). A Pearson correlation coefficient of *r*(108) = 0.87, *p* < 0.001 also indicted a strong association between test and retest mean confidence.

#### Individual differences and local, trial-level confidence

Examining correlations across task outcomes in the full sample (N = 3410), local confidence was slightly higher in individuals with greater task accuracy (*r*(3408) = 0.05, *p* = 0.008), in those with an increased mean dot difference (easier difficulty level on average) (*r*(3408) = 0.14, *p* < 0.001), and in those with faster reaction times (*r*(3408) =  − 0.08, *p* < 0.001). Of note, the large sample size in this study (N = 3410) may contribute observations of statistical significance, even for very weak associations (e.g., *r*(3408) = 0.05, *p* = 0.008 for the correlation between mean local confidence and mean accuracy). There was a strong correlation between local trial-level confidence and global round-level self-performance evaluations (*r*(3408) = 0.79, *p* < 0.001) (Fig. 3C), replicating the association reported in Experiment 1. When examining the effects of device type (Apple/Android), participants with Apple devices (n = 506) had higher mean confidence (*r*_pb_(3408) = 0.07, *p* < 0.001), higher mean global evaluations (*r*_pb_(3408) = 0.05, *p* < 0.001) and slower reaction times (*r*_pb_(3408) = 0.11, *p* < 0.001) compared to Android users (n = 2904). There was no association between device type and task accuracy (*r*_pb_(3408) = 0.01, *p* = 0.725), difficulty (*r*_pb_(3408) =  − 0.02, *p* = 0.352), or levels of educational attainment (χ^2^(1) = 0.08, *p* = 0.771). Android devices were more common among female (χ^2^(2) = 8.59, *p* = 0.003) and older participants (*r*_pb_(3408) =  − 0.24, *p* < 0.001).

Including sociodemographic factors in separate models, older adults (*r*(3408) = 0.06, *p* < 0.001), males (*r*(3408) = 0.09, *p* < 0.001) and those with lower levels of educational attainment (*r*_pb_(3408) = 0.10, *p* < 0.001) had significantly higher mean local confidence. Controlling for age, gender and levels of education in the model, participants with higher levels of anxious-depression had lower levels of mean confidence (β =  − 0.27, SE = 0.02, t =  − 13.41, *p* < 0.001, r^2^ = 0.05) while those with higher levels of compulsivity and intrusive thought had elevated mean confidence (β = 0.08, SE = 0.02, t = 3.97, *p* < 0.001, r^2^ = 0.004) (Fig. 3D). Although mean accuracy and device type were associated with mean confidence, including these as additional covariates in the model did not affect the significant association between confidence bias with anxious-depression (β =  − 0.26, SE = 0.02, t =  − 12.98, *p* < 0.001) or compulsivity and intrusive thought (β = 0.08, SE = 0.02, t = 3.94, *p* < 0.001).

After controlling for mental health dimensions, the effect of age on local confidence was no longer significant (β = 0.02, SE = 0.02, t = 1.04, *p* = 0.299), while the effects of gender (β = 0.09, SE = 0.02, t = 4.84, *p* < 0.001) and educational attainment (β = 0.28, SE = 0.03, t = 8.14, *p* < 0.001) held within this model. As the controlled effect of age on local confidence was not significant in the model, we examined which variables accounted for the significant correlation between age and local confidence. Specifically, accounting for anxious-depression in the model removed the significant effect of age on local confidence (β < 0.001, SE = 0.02, t = 0.03, *p* = 0.979).

#### Individual differences and global, round-level self-performance evaluations

Unlike local confidence, mean global self-performance evaluations were not significantly correlated with task accuracy (*r*(3408) = 0.03, *p* = 0.071), but like local confidence, global evaluations were higher in those who had an increased mean dot difference (easier difficulty level) (*r*(3408) = 0.12, *p* < 0.001), those who had faster response times (*r*(3408) = − 0.08, *p* < 0.001), and those with Apple devices (*r*_pb_(3408) = 0.05, *p* = 0.001).

Similar to local confidence, older adults *(r*(3408) = 0.06, *p* < 0.001), males (*r*(3408) = 0.15, *p* < 0.001) and those with lower levels of educational attainment (*r*(3408) = 0.05, *p* < 0.001) had significantly higher global self-performance evaluations, when included in separate models. Consistent with the local confidence results, those with higher levels of anxious-depression had lower global self-performance evaluations (β =  − 0.26, SE = 0.02, t =  − 13.28, *p* < 0.001, r^2^ = 0.05), and those with higher levels of compulsivity and intrusive thought had increase global evaluations (β = 0.04, SE = 0.02, t = 2.27, *p* = 0.023, r^2^ = 0.001), controlling for age, gender and education in the model (Fig. 3D). Similar to local confidence, the uncontrolled effect of age on global evaluations became non-significant when the transdiagnostic dimensions were included in the model specifically (β = 0.004, SE = 0.02, t = 0.23, *p* = 0.816).

#### Measuring metacognition in few trials

**Figure 4:**
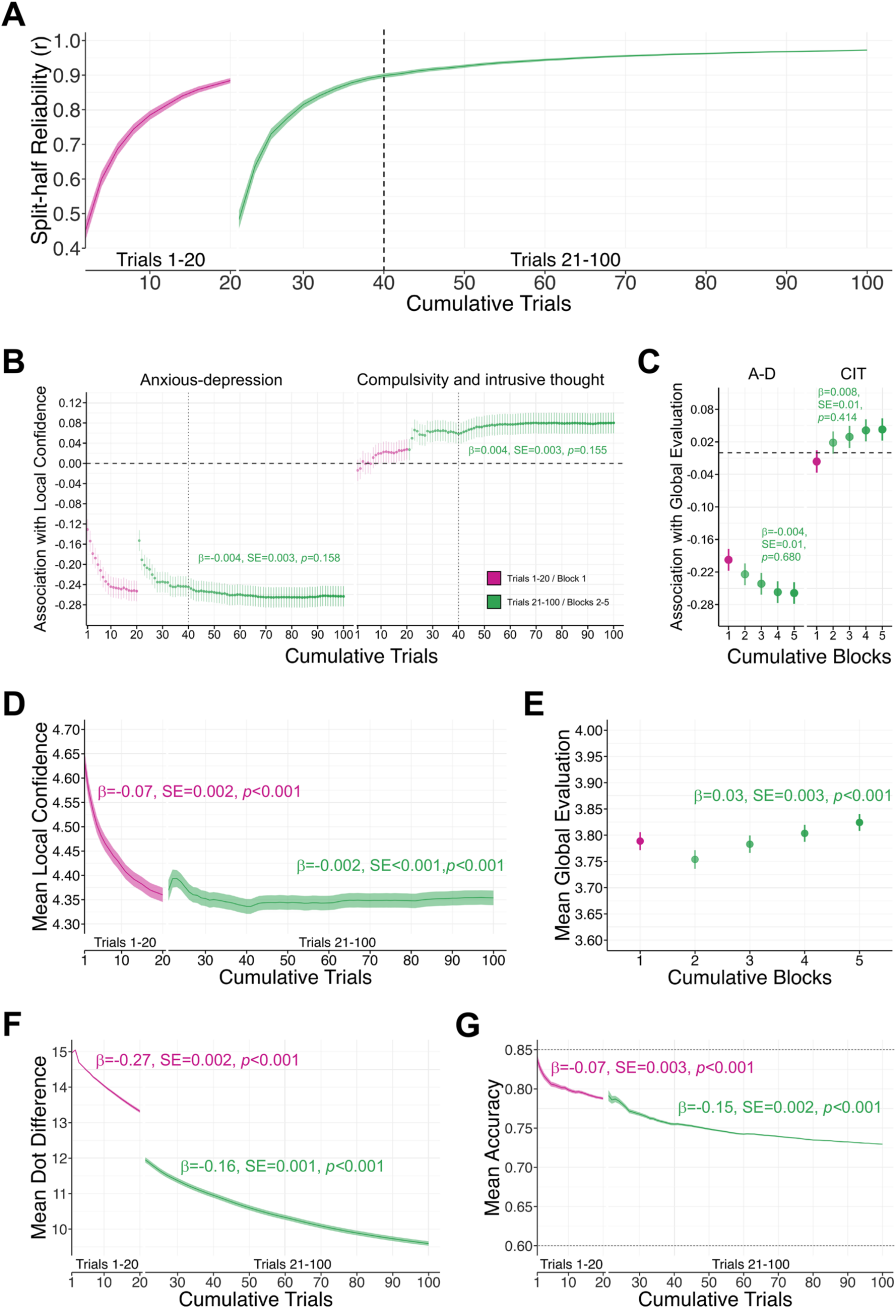
Impact of increasing trial number on mean confidence (N = 3388). β = standardised beta coefficient, SE = standard error, *p* = *p* value, *** = *p* < 0.001, ** = *p* < 0.01, * = *p* < 0.05 A-D = anxious-depression, CIT = compulsivity and intrusive thought. The error bars represent the standard error around the standardised beta coefficient. (**A**) Split-half reliability of mean local confidence was calculated separately for the initial 20 burn-in trials (pink) and the 80 main game play trials (green). Only 40 trials were required to estimate the split-half reliability of local confidence at *r* = 0.90. (**B**) The significant associations between mean local confidence with anxious-depression and compulsivity and intrusive was evident at 40 trials (vertical lines) and remained stable to 100 trials. (**C**) Following the burn-in block (pink error bar, block 1), there was no significant interaction effects of additional block number and transdiagnostic dimensions on mean global evaluation, indicating that associations with global evaluations remained stable with cumulative round-level ratings (green error bars, blocks 2–5). (**D**) While mean confidence significantly reduced during the burn-in period for the staircase (pink line, trials 1–20), confidence estimates were more stable across the remaining 80 main game trials (green line, trials 21–100). (**E**) Following the burn-in block (pink bar, block 1), mean global evaluations increased with cumulative blocks (green error bars, blocks 2–5). (**F**) Mean dot difference and (**G**) mean accuracy declined across the burn-in and then continued to decline across main game trials.

To examine the impact of trial number on estimates, we removed 22 participants that were missing data for at least one trial due to a software bug, leaving 3388 participants with 100 trials of confidence and task performance data. First we examined split half-reliability for local confidence (Fig. 4A). The exponential function fitted to the sensitivity curve of split-half reliability for main game trials accounted for a significant proportion of variance explained in local confidence ratings (r^2^ = 0.98). Internal consistency for mean confidence with the main game trials reached the 95% asymptote with the exponential model fitted to the split-half reliability values at just 40 trials (*r* = 0.90, 95% CI [0.89–0.90]) (Fig. 4A, vertical dashed line). Split-half reliability then plateaued and reached *r* = 0.97 (95% CI [0.97–0.97]) when based on the full 100 trials (Fig. 4A).

Figure 4B shows how the association between mean confidence with anxious-depression and compulsivity and intrusive thought changes with increasing trial number, controlling for age, gender, levels of education in the model. As 40 trials were required to optimise reliable estimates of local confidence, we examined the stability of clinical correlates with local confidence from 40 to 100 trials. The negative association between mean local confidence and anxious-depression was significant with 40 trials (β =  − 0.24, SE = 0.02, t =  − 12.13, *p* < 0.001) and with 100 trials (β =  − 0.26, SE = 0.02, t =  − 13.11, *p* < 0.001). The association between local confidence and anxious-depression remained stable from 40 to 100 main game trials, with no significant interaction effect of anxious-depression and trial number on mean confidence (β =  − 0.004, SE = 0.003, t =  − 1.41, *p* = 0.158) (Fig. 4B). Similarly, the positive association between local confidence and compulsivity and intrusive thought was evident at 40 trials (β = 0.06, SE = 0.02, t = 2.84, *p* = 0.005) and at 100 trials (β = 0.08, SE = 0.02, t = 3.90 *p* < 0.001), and remained stable from 40 to 100 main game trials (β = 0.004, SE = 0.003, t = 1.42, *p* = 0.155) (Fig. 4B). Therefore, clinical correlates with local confidence could be detected with only 40 trials. Excluding the first block’s rating, there was no significant interaction effect of block number with anxious-depression (β =  − 0.004, SE = 0.01, t =  − 0.41, *p* = 0.680) or compulsivity and intrusive thought (β = 0.008, SE = 0.01, t = 0.82, *p* = 0.414) on mean global evaluations, indicating clinical correlates with global evaluations were also stable across blocks (Fig. 4C). Clinical correlates with mean local confidence and global evaluations were also stable across the binned trials (see Supplementary Results).

Finally and for completion, we examined changes in the mean level estimates from the task over trials. In the first block, there was a significant decrease in mean confidence with the accumulation of trials (β =  − 0.07, SE = 0.002, t =  − 49.16, *p* < 0.001) (Fig. 4D, trials 1–20). For the subsequent 80 main game trials, mean confidence slightly decreased further, but this effect was small (β =  − 0.002, SE < 0.001, t =  − 4.05, *p* < 0.001) (Fig. 4D, trials 21–100). Excluding the first block rating, mean global evaluations, in contrast, slightly increased with the accumulation of blocks (β = 0.03, SE = 0.003, t = 10.09, *p* < 0.001) (Fig. 4E). As expected, the mean dot difference reduced throughout the first block (β =  − 0.27, SE = 0.002, t =  − 149.30, *p* < 0.001) and main game trials (β =  − 0.16, SE = 0.001, t =  − 273.6, *p* < 0.001), reflecting an increased in task difficulty with continued game play (Fig. 4F). Finally, accuracy significantly declined during the burn-in trials (β =  − 0.07, SE = 0.003, t =  − 24.79, *p* < 0.001) and continued to decline throughout the game (β =  − 0.15, SE = 0.002, t =  − 90.91, *p* < 0.001) (Fig. 4G). Despite this, accuracy remained firmly within the bounds of the upper and lower limits of the acceptable range (60–85%).

#### Measuring metacognition repeatedly with the 40-trial version of Meta Mind

**Figure 5:**
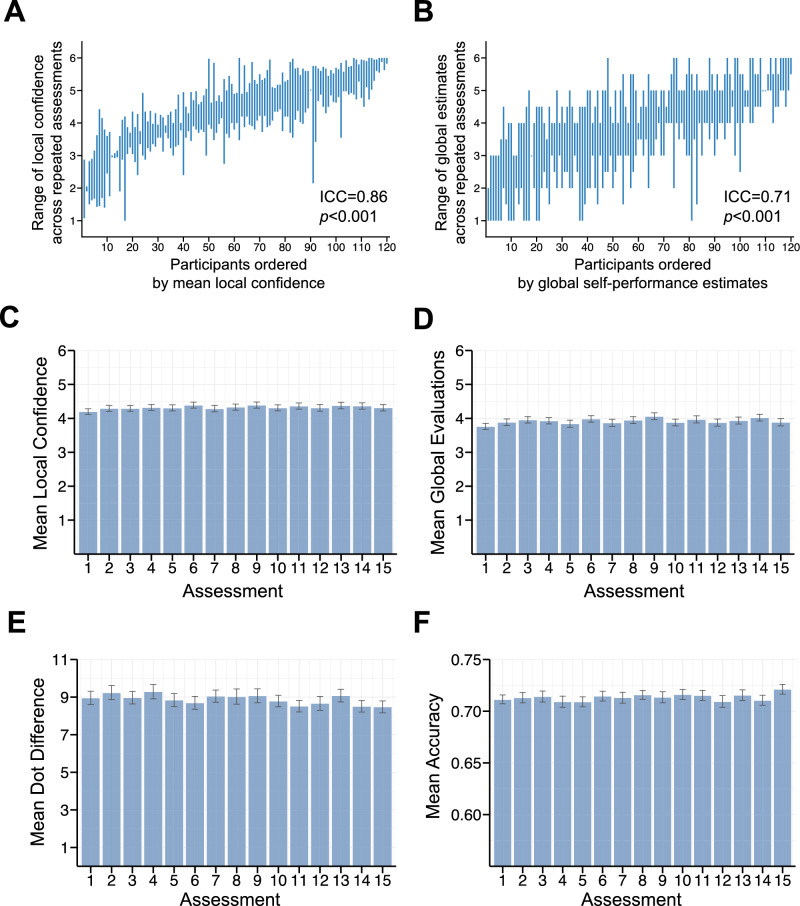
Measuring metacognition longitudinally (N = 120). ICC = intra-class correlation coefficient, SE = standard error, *p* = *p* value. Good test–retest reliability was evident for (**A**) mean local confidence and (**B**) global self-performance estimates among the N = 120 that played the abbreviated version of Meta Mind 15 times over 8 weeks. (**C**) Mean local confidence marginally increased, but (**D**) global self-performance evaluations did not significantly change when examining the impact of timepoint with binned assessments. (**E**) Task difficulty increased across the 15 assessment points, as indexed by a decrease in mean dot difference. (**F**) Due to low variance, the mixed-model across the 15 assessment points would not converge for mean accuracy. In a simpler comparison of 1st versus 15th session, there was no significant change in accuracy.

A sub-sample of 120 citizen scientists played an abbreviated, 40-trial version of Meta Mind 15 times over an 8-week period, with a median time interval of 2 days (SD = 1.06) between games played. Among this subsample, test–retest reliability for local confidence was good across the 15 assessment points (ICC (A,1) [CI] = 0.86 [0.83, 0.89], *p* < 0.001) (Fig. 5A). Similarly, global self-performance estimates had good reliability across the 8-week period (ICC (A,1) [CI] = 0.71 [0.65, 0.76], *p* < 0.001) (Fig. 5B). To characterise any potential practice effects, we ran linear mixed-model analyses to examine the fixed effect of assessment timepoint on mean local confidence, mean global evaluations, mean accuracy and mean difficulty, with participants as a random factor. There was a marginally significant increase across assessment points in mean confidence (β = 0.02, SE = 0.008, t = 1.97, *p* = 0.050) (Fig. 5C), but no significant change in global self-performance estimates (β = 0.004, SE = 0.01, t = 0.35, *p* = 0.727) (Fig. 5D). Mean dot difference significantly decreased across assessment points (β =  − 0.04, SE = 0.01, t =  − 2.51, *p* = 0.012), reflecting an increase in task difficulty across time as participants became better at the perceptual discrimination task (Fig. 5E). Accuracy was staircased, and as a result the values were highly stable across days (Fig. 5F). Indeed, due to low variance, the mixed-model across the 15 assessment points would not converge for the analysis of mean accuracy. As we could not fit this model, we instead compared first versus 15th assessment, and found there was no significant change in accuracy when comparing the assessment timepoints (β = 0.19, SE = 0.12, t = 1.60, *p* = 0.110).

## Discussion

There is growing interest in the study of metacognition in psychiatric populations^3,10^, with well-replicated observations of reduced confidence in those with higher levels of anxious-depression and elevated confidence in those endorsing symptoms of compulsivity and intrusive thought^12–16^. But progress in understanding the mechanisms underlying these biases in transdiagnostic psychiatric dimensions has been slower, in part due to overreliance on cross-sectional study designs, paid participants from crowdsourced platforms and unknown psychometric properties of mainstay tests. In this study, we aimed to support a move towards repeated within-person and remote assessment by developing a brief and reliable task to measure metacognition via a smartphone application among citizen scientists.

In a considerably abbreviated smartphone task, metacognitive bias had acceptable convergent validity, as mean confidence was moderately correlated across traditional and smartphone tasks. Split-half reliability for metacognitive bias was excellent and empirically stabilised after 20 game trials, which included the burn-in period, corresponded to requiring 40 trials in total. With both the 100- and 40-trial versions of Meta Mind, metacognitive bias had good test–retest reliability, indicating that metacognitive estimates for each individual were highly stable across time. This is consistent with previous findings of strong test–retest reliability for confidence ratings in a traditional visual metacognitive task, with as few as 50 trials^39^. This high level of reliability in our study contrasts with recent reports that other cognitive tasks under study in the field of computational psychiatry suffer from poor reliability^33^. In contrast, prior work suggests that self-report measures are considerably more reliable than behavioural readouts alone^30,31^. This facet of our task, coupling self-report with the experimental control of a behavioural test^32^, may explain the high reliability that we observed. This sort of ‘hybrid ‘self-report cognition’ task may be one way to bridge the recently discussed reliability gap in computational psychiatry. This approach may be particularly suited for studying the mechanisms of biases in thinking and feeling specifically, which may only noisily lead to downstream changes in behaviour on a task. We showed that task accuracy was highly stable across multiple sessions, indicating there was no significant practice effects with Meta Mind. This was in line with the task design, as stability in task performance is maintained by adjusting in task difficulty, which increased across sessions. In addition to practice factors, affective state can be a source of significant temporal variability in cognitive task performance^35^. However, we did not measure any changes in affective state or psychiatric symptoms across time. Given that confidence bias is state-dependent^13^ and changes over a period of days^18^, we would expect stable metacognitive abilities in our sample to coincide with stability in psychiatric states across time. An interesting target of future research may be to uncover the dynamic nature of the relationship between metacognition and mental health and as it fluctuates with mental state. This could be achieved by adopting Meta Mind as a clinical tool to monitor these fluctuations across various timescales (e.g., over hours, days, weeks). Assessing these dynamic interactions repeatedly over time is the next step to test causal models of metacognition and psychopathology.

As a further demonstration of validity, we replicated the established patterns of local metacognitive biases across transdiagnostic psychiatric dimensions^12–16^. As the associations between metacognitive bias and psychiatric dimensions are replicable across different task types, including a learning task^14^ and for general knowledge^15^, this would suggest that our findings on metacognition are not task-dependent, and would translate to other decision-making tasks, regardless of the cognitive facet. Specifically, those with higher levels of anxious-depression had lower confidence, while those with higher levels of compulsivity and intrusive thought had elevated confidence in their performance. We additionally found associations between psychiatric dimensions and global evaluations, which were analogous to the local confidence level results. Anxious-depression was associated with reduced global evaluations of performance, which is consistent with prior work showing widespread biases across the metacognitive hierarchy, from low-level perceptual decisions up to notions of self-worth^3,16^. Conversely, greater levels of compulsivity and intrusive thought were associated with higher global estimates^16^, but were only marginally significant with a negligible effect size. This could suggest that overconfidence in compulsivity and intrusive thought manifests more at the trial-level, due to specific learning biases for local decision-making. Indeed, this was observed in a prior study using a reinforcement learning task, where compulsivity, but not anxious-depression, was linked to a failure to use trial-level feedback to update metacognitive bias^14^.

While the effect of compulsivity and intrusive thought on local confidence bias was significant, this was weaker than the effects reported in prior studies that used dot discrimination metacognitive tasks^12,13,15,16^. This may be because we used a reduced set of questions that a prior study demonstrated were highly predictive of scores^41^, based on the full 209 questionnaires items from the original paper^42^. It is therefore possible that this decreased the sensitivity to detecting overconfidence in compulsivity and intrusive thought. The reduced set omitted items from the schizotypy scale for example, which might be important for capturing positive symptoms that are linked to overconfidence bias^43^. Given this, future studies with smaller sample sizes should consider using the full questionnaire items to have sufficient power to detect the associations between local confidence and psychopathology^12,13,15,16^. Although we provide evidence for Meta Mind’s convergent validity, this is specifically within the visuo-perceptual domain. While there is evidence that visuo-perceptual metacognition generalises across domains (e.g., convergence with confidence in general knowledge^15^), the validity of Meta Mind as a measure of domain-general metacognition should be further investigated by comparing Meta Mind to metacognitive tasks in alternative domains (e.g., general knowledge, memory, or even other perceptual modalities).

It must be noted that the distinction between global and local assessments was subtle in Meta Mind. Across both experiments, there was overall a strong cross-sectional correlation between local confidence and global evaluations. While this is in line with other studies^16,44^, this strong association could mean that our measure of global evaluations (task-level rating) might be too proximal to the local level to reflect its aggregation over time. Future research should consider using alternative measures for global metacognition, such as task-level choices, which may be less proximal to local confidence ratings^2,16^. Although we included measures of local confidence and global performance evaluations within the task, a limitation of the current study is that we did not collect a measure of global self-beliefs. Global self-beliefs, such as self-esteem, are higher-order stable traits that are thought to be partially informed by local and global confidence^3^. Recent work using this hierarchical framework demonstrated that self-esteem, a form of global self-belief, is lower in anxious-depression and to a lesser extent, compulsivity and intrusive thought^16^. This differs markedly from observations at the bottom of the hierarchy—i.e., local confidence—where opposing patterns of association are consistently observed for these transdiagnostic traits. Further, discrepancies in how local confidence and global self-beliefs interact were recently observed in a parallel paper using the same smartphone task from the present study (Meta Mind)^44^. In this study, self-esteem in problem gamblers was found to be correlated with local confidence, but no such correlation was observed in comparison subjects who did not gamble^44^. To unpack this, future studies with large samples could consider including measures of high level self-beliefs, to investigate the contemporaneous and temporal relationships between the various hierarchical levels of metacognition and psychiatric dimensions, with particular focus on how they develop and interact over time.

The association between local confidence and transdiagnostic dimensions could be detected rapidly, with 40 trials required to detect stable estimates of underconfidence in anxious-depression and overconfidence in compulsivity and intrusive thought, respectively. Considering this with split-half reliability findings, valid and precise estimates of local metacognitive biases can be obtained with as few as 40 trials. Although global evaluations of self-performance increased with cumulative ratings, the association between global evaluations of self-performance and anxious-depression could be detected with a single rating and did not increase with more ratings. The weaker association between global evaluations and compulsivity and intrusive thought was also stable across block ratings.

In contrast to metacognitive bias, we found that metacognitive efficiency was neither valid nor reliable in Meta Mind. While M-Ratio is the dominant standard measure of metacognitive efficiency, estimates are dependent on trial number^39,45^. Unacceptable internal consistency for M-Ratio with Meta Mind is consistent with previous findings of poor split-half reliability for M-Ratio with trial numbers below 100^39,45^. This raises questions over the continued use of this metric in individual differences research, despite the widespread use of lower trial numbers than recommended. Even with 400 trials, test–retest reliability of M-Ratio was found to be very low^39^, indicating that M-Ratio estimates may lack stability, as opposed to being ‘unreliable’ per se. To account for any instability in M-Ratio estimates, future studies should account for potential sources of temporal variability, such as state-like changes^33,35^. Overall, our data lend support to the idea that researchers should carefully choose a reliable measure, suitable for the task design and appropriate to the inferences about individual differences in metacognitive efficiency they aim to make.

Examining sociodemographic factors, males and individuals with lower levels of educational attainment had higher local confidence consistent with previous findings^13^. With respect to global evaluations of performance, the direction of effects was the same but interestingly, effect sizes for gender were much larger for global compared to local and the opposite was observed for educational attainment. While uncontrolled analyses suggested that older adults had higher local confidence and global evaluations, this effect was also no longer present when we accounted for psychiatric dimensions in the model. While higher local confidence and global evaluations have been previously reported among younger adults^46^, the true direction of these effects may have been obscured, as mental health related factors were not accounted for. Indeed, prior inconsistent findings as to whether metacognitive abilities vary according age^12,13,46,47^ or gender^12,13,48,49^ may be due to a lack of consideration of psychiatric dimensions as potential covariates in the model. Local confidence and global evaluations varied according to device type, indicating software and hardware variability should be considered when utilising smartphone-based research methods^50^. This raises new challenges for research, as device types, far from being randomly allocated in the population, are associated with important socio-demographic characteristics and cognitive capacities we wish to study^51^.

In terms of basic game mechanics, relative to the traditional task, Meta Mind was considerably shorter at 7.86 versus 21.50 min. Mean accuracy and confidence were also higher on average for Meta Mind. Trial-by-trial analysis revealed that accuracy steadily declines with additional trials, suggesting that although the staircase procedure maintained mean accuracy within a narrow and desired range (74% correct on average), more trials would decrease it somewhat further before reaching an asymptote. While task accuracy was weakly associated with local confidence, controlling for accuracy in our model did not affect the association between metacognition and transdiagnostic dimensions. The staircase functioned optimally across assessments, as task accuracy was stable with repeated game play. Relative to a metacognitive questionnaire, the main benefit of Meta Mind as an experimental task is the assessment of confidence while controlling for performance accuracy across participants. For example, an individual could report low confidence in their visual abilities on a self-report scale, but this may be an accurate appraisal of their abilities if they indeed have poor vision. With Meta Mind, we can determine true bias towards lower or higher confidence, controlling for visual task performance. Similar to accuracy, dot difference steadily declines throughout the task. This may reflect the design of Meta Mind, a relatively easier task, especially during the initial trials. Readjusting the dot difference of the starting trials to be more difficult may stabilise task difficulty and accuracy over fewer trials.

Overall, the study provided further support for utilising a combination of smartphone-based methods and a citizen scientist framework to conducting large-scale mental health research. The sample size of this study is the largest to date that examined how metacognitive abilities vary with psychopathology. With that, data quality was excellent. We employed a battery of checks for our task and questionnaire data^38^, and less than 2% of participants were excluded for careless or inattentive responses. This is in stark contrast to recent reports with paid crowdsourced participants^20^. As citizen scientists were not financially incentivised to complete Meta Mind, their intrinsic motivations may translate to conscientious participation. Although Meta Mind was designed to be more enjoyable and engaging that the traditional task, we did not collect any qualitative feedback from participants on of playing Meta Mind. While the majority of citizen scientists that completed the tutorial trials went on to play the game in full, we did not have a direct indicator of task enjoyment.

Relative to the traditional task, Meta Mind was much shorter, but was able to replicate previous finding of disruption to metacognition at different levels of the hierarchy across psychiatric dimensions. This brief gamified task demonstrated validity and high reliability even with as little as 40 trials, allowing for the precise and rapid measurement of metacognitive bias. This study also provided more general support for utilising smartphone-based methods and a citizen science framework, to scale up and speed up cognitive science. The next frontier is to uncover the dynamic interactions between metacognition and psychopathology, which can be achieved by using Meta Mind as a tool to monitor within-person disruptions to metacognitive stability over time.

## Methods

### Experiment 1

#### Participants

Individuals in Experiment 1 were recruited by convenience sampling; through word of mouth, social media, online forums and university mailing lists. Participants were included if they were over the age of 18 and had access to a computer (desktop/laptop) and smartphone (Apple/Android device). Of the N = 116 participants that consented to participate, N = 52 met inclusion criteria and fully completed Meta Mind on their personal smartphone device and the traditional metacognitive task on in a web-browser. The sample had a mean age of 23.62 (SD = 7.96), was mostly female (n = 39, 75.00%), living in Ireland (n = 49, 94.20%), and had obtained at least secondary school level education (n = 50, 96.10%) (Table 1). Participants were paid €10 for taking part. Power analysis was based on a prior study that gathered data on the same traditional browser-based metacognitive task at baseline and 4 weeks later. This study reported an ICC of 0.73 for metacognitive bias, with 95% confident interval = 0.67–0.77^13^. We anticipated that the convergent validity (Pearson correlation) estimates when comparing tasks in this study might be smaller, given differences across the tasks in length, instructions, interface, graphics and confidence rating scales. Conservatively, we powered our study to detect a medium effect size (*r* = 0.40) with 0.80 power, requiring 46 participants.

**Table 1:** Baseline sociodemographic characteristics of participants in experiment 1.

Characteristic	Paid participants (N = 52)
Gender, No. (%)	
Male	13 (25.0)
Female	39 (75.0)
Age, M (SD)	23.62 (7.96)
Country of residence, No. (%)	
Ireland	49 (94.2)
United Kingdom	3 (5.8)
Education, No. (%)	
Primary level	1 (1.9)
Secondary level	32 (61.5)
Undergraduate degree	18 (34.6)
Above undergraduate degree	1 (1.9)
Ethnicity, No. (%)	
White or Caucasian	45 (86.5)
Asian or Pacific Islander	4 (7.7)
Multiracial or biracial	3 (5.8)
Employment status, No. (%)	
Unemployed	27 (51.9)
Part-time employed	23 (44.2)
Full-time employed	2 (3.8)

#### Procedure

Participants accessed the study information and an electronic consent form via Qualtrics, the link for which was embedded in the study advertisement. After providing consent, participants provided their email address and the following sociodemographic information: age, gender, level of education, country of residence, ethnicity and employment status. Participants that met inclusion criteria received an email from the research team containing their unique Study ID, with instructions on how to download the app and complete Meta Mind and a hyperlink to access the traditional metacognitive task in their browser. The email specified the sequence that each participant should complete the tasks, which was counterbalanced across the sample.

##### Traditional metacognitive task

The traditional metacognitive task was a visuo-perceptual decision-making task, which has been previously described^13^. The task could be completed by participants via a web-browser on their personal computer (Fig. 1A). On each trial, participants were shown a fixation cross for 1000 ms (ms), followed by two sunflowers, positioned on the left and right of the screen for 300 ms. After the sunflowers disappeared from the screen, participants had unlimited time to make a judgement about which contained more seeds. The chosen sunflower was highlighted for 500 ms, but no feedback on accuracy was provided. Participants then rated their confidence in each judgement, on a scale from ‘Guessing’ to ‘Certain’. There was a total of 210 trials, divided equally into five blocks. Accuracy was controlled using a ‘two-down one-up’ staircase procedure, in which the task became easier (i.e. a larger seed difference between sunflowers) after each incorrect response and more difficult (i.e. a smaller seed difference between sunflowers) after two consecutive correct responses. This maintained objective performance across all participants between 60 and 85% correct, which ensured that estimates of confidence were not confounded by performance differences, and confidence biases can be accessed when accuracy does not vary across individuals. The first 25 trials participants experienced were in tutorial format and used as burn-in (i.e., to stabilise accuracy), and thus not used in the calculation of behavioural metrics like mean confidence. One sunflower was always half-filled (313 dots out of 625 positions), while the other box contained an increment of + 6 to + 81 dots compared to the standard. Changes in difficulty (seed differences between stimuli) were calculated in log-space, with a starting log difference of 4.2 (+ 70 dots). Differences in step size changed by ± 0.4 for the first five trials, ± 0.2 for the next five trials and ± 0.1 for the remainder of the task. Seed differences on each trial could range from as few as six dots (1.79 in log-space—the hardest to discriminate) to as many as 81 dots (4.39 in log-space—the easiest).

##### Meta Mind

Participants downloaded the smartphone app *Neureka* and entered their unique Study ID to their app profile, which differentiated their data from that of unpaid citizen scientists. In Meta Mind, players travel in a spaceship through the brain and make a series of choices based on stimuli they meet along the way (Fig. 1B). At the start of each round, players are presented with a fact about how brain health may be impacted by factors like sleep, diet and spending time in nature. Players are then instructed to navigate their ship to the stimuli containing more dots. On each trial, players presented trial-by-trial with pairs of moving icons representing that brain health fact (e.g. brains), that differ in the number of dots contained within them. As icons descend down the screen, players must select the icon with more dots, touching the screen to navigate their ship left or right to collide with the icon. After players make a choice, they then rate their confidence in the accuracy of their choice. After 20 trials, players evaluate their overall accuracy on that round, forming a ‘global’ self-performance evaluation based on round performance. Although no direct feedback on accuracy is provided, for gameplay reasons, participants are informed that the task will get harder when they get better at it and at the end of each round, the difficulty level for the next round is indicated to participants.

Meta Mind has three instructed tutorial trials, which are followed by 100 game play trials, divided into five rounds of 20 trials. The first 20 trials of game play are used to burn-in the ‘two-down one-up’ staircase, which maintains the average accuracy within a range of 0.60–0.85, as per the traditional task. The first 20 trials are not used for the calculation of task outcomes, leaving the 80 subsequent trials for analyses. On each trial, there is always one stimulus with 100 dots, randomly presented on left- or right-hand side of the screen. The minimum number of dots in the comparison icon is 101 and the maximum number of dot positions in the comparison stimuli is 149, which still left areas of unfilled space within the stimuli. As per the traditional task, change in dot differences is calculated in a log-space. The difficulty ranged from level 1 (easiest level, dot difference of 49 and a corresponding log value of 3.9) to level 26 (most difficult level, dot difference of 1 and a corresponding log value of 0.4). The tutorial trials all have a difficulty level of 1 and the first game play trial has a difficulty level of 13 (dot difference of 15 and a corresponding log value of 2.7), which changes based on trial-level accuracy over the subsequent 100 trials.

Ethical approval for both experiments was obtained from the Research Ethics Committee of School of Psychology, Trinity College Dublin (Approval ID: SPREC072019-01). All methods were performed in accordance with the relevant guidelines and regulations. Both experiments only included adults over the age of 18.

#### Data preparation and analysis

##### Behavioural outcomes and exclusions

Explicit confidence judgements are the conventional measure of metacognition in experimental tasks, required to evaluate meta-representations of the self^7^. For the traditional metacognitive task and Meta Mind, our primary outcome measure was metacognitive bias, calculated as mean confidence across trials. For both tasks, confidence on each trial was rated on a 6-point numeric scale, where ‘Guessing’ (traditional task)/‘Low’ (Meta Mind) = 1, and ‘Certain’ (traditional task)/‘High’ (Meta Mind) = 6. As shown in Fig. 1, the text on the scales, but not the corresponding numbers, were presented to participants. Metacognitive efficiency (M-Ratio) was also calculated, which is the ratio of metacognitive sensitivity to mean accuracy, where sensitivity is the extent to which confidence ratings discriminate between correct and incorrect trials. M-Ratio, the gold-standard measure of metacognitive efficiency^39^, was calculated in a hierarchical Bayesian framework (single-subject estimations) using the freely available HMeta toolbox^52^, http://github.com/smfleming/HMM, accessed June 2022. An M-Ratio value of 1 indicates that confidence was fully informed by accessing the total perceptual information available. ‘Global’ self-performance evaluations was calculated as the mean of round-level accuracy ratings across the four game rounds (every 20 trials). Self-performance evaluations were rated on a scale at the end of each block, from ‘Change Level (1)’ to ‘Perfect (6)’. Task difficulty was measured as the mean seed/dot difference across trials, where more difficult trials had a smaller difference between stimuli. Mean reaction time to stimulus choice across trials was measured in seconds and task accuracy was calculated as the mean proportion of correct responses across trials. Given the remote, online study design, participants had unlimited time to complete the tasks, and could do so across multiple days. Therefore, to estimate game completion time, we only considered completion times under 60 min (removing n = 5 for Meta Mind and n = 2 for the traditional task from this specific metric). Of N = 116 participants recruited, N = 62 completed the traditional task and the N = 59 participants that played Meta Mind, with N = 52 completing both. We employed a number of established exclusion criteria to ensure high data quality from the metacognitive task^13^. Due to a software bug, data for single trials on both tasks were missing choice response time for a small proportion of total trials (mean percentage of trials missing data was 0.02% for each task). These trials with missing data were discarded when calculating behavioural outcomes. For both tasks, participants who selected the right or left stimuli on more than 95% of trials or who had a mean accuracy < 0.60 or > 0.85 were to be excluded, but no participants met these criteria.

##### Statistical analysis

Linear regression analyses were conducted with task type (Meta Mind or traditional task) as the independent variable to determine the effect of task type on the following task performance characteristics as separate dependent variables: time to complete, mean confidence, M-Ratio, mean accuracy and mean difficulty. Convergent validity, the extent to which two tasks measure the same underlying construct, was assessed using Pearson product moment correlation analyses for (1) mean confidence and, (2) M-Ratio. Split-half reliability, the consistency across each half of a measure, was assessed through Pearson product moment correlation analyses between odd and even trials on (1) Meta Mind and, (2) the traditional task, consistent with prior publications^39,45^. To determine the effect of task order on performance characteristics, linear regression were conducted with the order of task (Meta Mind being completed first or second) as the independent variable and the following separate dependent variables: mean confidence, M-Ratio, mean accuracy and mean difficulty (for Meta Mind/the traditional task). To evaluate the association between levels of the metacognitive hierarchy, Pearson correlation analysis were used to correlate local (trial-level) mean confidence with global (round-level) mean self-performance evaluations.

### Experiment 2

#### Participants

Of the 5997 general users of the Neureka app that completed the Meta Mind tutorial, 3776 (62.96%) played the full 100 trials between December 2021 and September 2023. After applying exclusion criteria (detailed below), N = 3410 individuals were retained for analyses. Participants were primarily female (n = 2401, 70.41%), with a mean age of 50.77 (SD = 13.88), were living in the United Kingdom (n = 2392, 70.15%) and had completed at least undergraduate level education (n = 2082, 61.06%) (Table 2). A power analysis was carried out using effect sizes from a previous study examining cross-sectional associations between metacognition and anxious-depression, and compulsivity and intrusive thought^12^. Sample sizes of N = 454 and N = 332 respectively were required to detect these associations with 80% power (linear regression analyses, two-tailed test). Therefore, the sample was well-powered to detect the association between metacognition and transdiagnostic psychiatric dimensions.

**Table 2:** Baseline sociodemographic characteristics of participants in experiment 2.

Characteristic	Unpaid participants (N = 3410)
Gender, No. (%)	
Male	936 (27.45)
Female	2401 (70.41)
Other gender identity	73 (2.14)
Age, M (SD)	50.77 (13.88)
Country of residence, No. (%)	
United Kingdom	2392 (70.15)
United States	474 (13.90)
Ireland	299 (8.77)
Other	245 (7.18)
Education, No. (%)	
Below undergraduate level	1328 (38.94)
Completed at least undergraduate level	2082 (61.06)

#### Procedure

##### Neureka

Neureka, a nonprofit smartphone application developed and managed by the Gillan Lab at Trinity College Dublin, is available to download for free on Apple or Android phones through the Apple Store or Google Play Store. Since being launched in 2020, Neureka has over 23,000 registered users across 139 countries as of September 2023. The Neureka project aims to enrol members of the general public in scientific research, by voluntarily playing games that tap into distinct cognitive processes underlying brain health^27^. After downloading Neureka, users provide informed consent and provide the following sociodemographic information upon registration: age, gender, levels of education and country of residence.

##### Meta Mind

Citizen scientists were able to play Meta Mind in Neureka by either completing (1) Meta Mind (n = 1130, 33.14%), a single session science challenge consisting of the game plus mental health questionnaires, or (2) another science challenge called ‘Brain Changer’ (n = 2280, 66.86%), a repeated session science challenge which includes a full version of Meta Mind (100 trials) and the same questionnaire to be completed on day 1, and is then followed by an abbreviated version of Meta Mind (reduced to 40 trials) bi-daily for 8 weeks alongside another bidaily cognitive test and daily self-report measures, which are not the topic of the present study. The design of Meta Mind was identical for all participants, regardless of which challenge was used to access the game. Of the 3410 participants that completed Meta Mind and the questionnaires described below, 110 played the 100-trial version Meta Mind twice within 30 days, once in Brain Changer and once in the stand-alone challenge. An additional 120 played an abbreviated, 40-trial version of Meta Mind 15 times across 8 weeks. These sub-samples of participants were used to examine the test–retest reliability of Meta Mind.

##### Self-report psychiatric questionnaires

Participants completed 49 items taken from six self-report questionnaires that assess a variety of psychiatric symptoms, including depression (Zung Self-Rating Depression Scale)^53^, trait anxiety (State Trait Anxiety Inventory)^54^, impulsivity (Barratt Impulsiveness Scale 11)^55^, obsessive–compulsive disorder (Obsessive–Compulsive Inventory–Revised)^56^, eating disorders (Eating Attitudes Test)^57^, apathy (Apathy Evaluation Scale)^58^ (see Supplementary Methods for the full list of items). These items were chosen based on a previous study that demonstrated the original set of 209 items used to generate the anxious-depression and compulsivity and intrusive thought factors could be reduced to 49^41^. In Brain Changer, the questionnaire items were presented to participants before the Meta Mind game. In the stand-alone Meta Mind challenge, items were completed by participants after playing the game.

#### Data preparation and analysis

##### Behavioural outcomes and exclusions

 All behavioural measures were the same as in Experiment 1, except we did not examine metacognitive efficiency because it exhibited poor psychometric properties in Experiment 1 and was not previously associated with transdiagnostic dimensions cross-sectionally^12,15,16^. In total, 3776 citizen scientists completed all 100 trials of Meta Mind. Data for single trials were excluded when choice response time were missing, due to a software bug (mean percentage of trials removed was 0.01%). No participants selected the right or left stimuli on greater than 95% of trials, but 12 (0.32%) participants had mean accuracy below 0.60 or above 0.85. N = 3764 participants therefore progressed to the next step.

##### Transdiagnostic psychiatric dimensions and exclusions

Individual scores on dimensions of anxious-depression and compulsivity and intrusive thought were calculated by multiplying each of the 49 individual questionnaire responses by the corresponding weights from a previously published regularised regression model^41^ trained to predict the original factors^42^. Dimension scores were scaled to centre on zero, with higher scores indicating higher levels of transdiagnostic psychopathology. Of the 3764 participants with adequate Meta Mind data, 3442 (91.45%) completed all the questionnaire items. To determine the proportion of careless/inattentive responders on the self-report clinical questionnaires, we included a ‘catch’ question that was embedded in the impulsivity scale (*I competed in the 1917 Summer Olympics Game*)^38^. Thirty-two (0.93%) participants did not respond ‘Never’ to this question, and were subsequently excluded from analyses, leaving 3410 participants that were retained for statistical analyses.

##### Statistical analysis

Test–retest reliability of local metacognitive bias was evaluated using the ICC (two-way mixed-effects model, absolute agreement, single rater^40^) of mean local confidence among the sub-sample of individuals that played Meta Mind twice. ICC values between 0.50 and 0.75 indicate moderate reliability, values between 0.75 and 0.90 have good reliability, and values greater than 0.90 have excellent reliability^40^. To determine the association between levels of the metacognitive hierarchy, we used Pearson correlation analysis to correlate local (trial-level) mean confidence with global (round-level) mean self-performance evaluations. To examine simple effects, we used correlation (Pearson and biserial) analyses to test for relationships between metacognition (local/global) with task outcomes (mean accuracy, difficulty and reaction time), sociodemographic factors (age, gender and education) and device type (Apple/Android). To examine the relationship between metacognition (local/global) and transdiagnostic psychiatric dimensions, we included anxious-depression and compulsivity and intrusive thought as independent variables within the same model, with age, gender and levels of education as covariates.

To examine the effect of increasing trial number on mean confidence estimates, we firstly calculated mean confidence step-wise, cumulatively across the first 20 trials (the burn-in period). That is, we calculated confidence on trial 1, then the average of trials 1 and 2, and so on until all 20 trials were averaged over. We then repeated this for the main game trials after the burn-in period, calculating mean confidence from the 21st trial and then increasing data with every subsequent trial, until the 100th trial. Linear mixed-model regression analyses were conducted to examine the effect of increasing trial number, with participants as random effects, on mean local confidence, mean global evaluations and mean accuracy for the burn-in trials and main game play trials separately. We also ran linear mixed-model analyses to examine the fixed effect of longitudinal assessment timepoint (15 in total) on mean local confidence, mean global evaluations, mean accuracy and mean difficulty, with participants as a random factor. The split-half reliability for mean local confidence was calculated using Pearson correlation analyses of odd and even trials, from the first 2 trials in bins of 2 trials until 100 trials (for the burn-in period and main game trial separately). We fit an exponential model to the area under the curve (AUC) sequence of split-half reliability values across main game play trials and calculated the minimal number of trials required to reach the asymptotic limit of split-half reliability for local confidence (95% of its asymptote across trial bins)^59^. To determine the minimal number of cumulative trials required to detect the association between metacognition and psychiatric dimensions, we examining the interaction effect of psychiatric dimension and trial number on mean local confidence/global evaluations, controlling for age, gender and level of education (across the burn-in trials and main game trials separately). An interaction effect with *p* > 0.05 for this analysis would indicate that the association between metacognition and psychiatric dimensions was not dependent on the trial number (i.e., the association was stable across trials).

For all tests in Experiment 1 and 2, statistical significance was defined as* p* < 0.05, with two-tailed* p* values used. Adjustments for multiple comparisons were not conducted. For regression analyses, all the dependent variables and continuous independent variables were z-scored before entering the models as to obtain standardised (i.e. comparable) regression coefficients. Gender was coded numerically (male = 1, female =  − 1, other = 0).

## Code and data availability

The R analysis scripts are available at https://osf.io/uba2d/. Access to the task and questionnaire data is restricted due to security reasons of sensitive data owned by Trinity College Dublin. Researchers may access the data by completing and submitting a Data Request Form for Research Purposes at https://osf.io/uba2d/.

### Supplementary Information

Supplementary Information.
