# Supplementary material for: Reliable, rapid, and remote measurement of metacognitive bias
Source: Sci Rep. 2024 Jun 28;14:14941. doi: 10.1038/s41598-024-64900-0 (PMC11213917; doi:10.1038/s41598-024-64900-0)
Supplement: Supplementary file 1 — Supplementary Information. [file 41598_2024_64900_MOESM1_ESM.pdf]

Additional File 1.
Supplementary Information

Reliable, rapid, and remote measurement of metacognitive bias

Celine A Fox[1,2,*], Abbie McDonogh[1], Kelly R Donegan[1,2], Vanessa Teckentrup[1,2], Robert J Crossen[1], Anna K Hanlon[1,2], Eoghan Gallagher[1,2], Marion Rouault[3], Claire M Gillan[1,2,4]

[1]Department of Psychology, Trinity College Dublin, Dublin, Ireland
[2]Trinity College Institute for Neuroscience, Trinity College Dublin, Dublin, Ireland
[3]Paris Brain Institute (ICM), Centre National de la Recherche Scientifique (CNRS), Paris, France
[4]ADAPT Centre for Digital Technology, Trinity College Dublin, Dublin, Ireland

*CAF is the corresponding author. E-mail address: foxce@tcd.ie

SUPPLEMENTARY METHODS

Self-report psychiatric questionnaires items

Participants completed the following 49 items taken from six self-report questionnaires that assess a variety of psychiatric symptoms[1], including depression (Zung Self-Rating Depression Scale)[2], trait anxiety (State Trait Anxiety Inventory)[3], impulsivity (Barratt Impulsiveness Scale 11)[4], obsessive-compulsive disorder (Obsessive-Compulsive Inventory-Revised)[5], eating disorders (Eating Attitudes Test)[6], apathy (Apathy Evaluation Scale)[7]:

Apathy Evaluation Scale (AES)

2. I get things done during the day. Reverse
7. I approach life with intensity. Reverse
17. I have initiative. Reverse
18. I have motivation. Reverse

AES response scores:
Not at all characteristic (1) gets a zero
Slightly characteristic (2)
Somewhat characteristic (3)
Very characteristic (4)

Barratt Impulsiveness Scale (BIS)

1. I plan tasks carefully. Reverse
6. I have "racing" thoughts.
9. I concentrate easily. Reverse

13. I plan for job security. Reverse
14. I say things without thinking.
15. I like to think about complex problems. Reverse
17. I act "on impulse".
20. I am a steady thinker. Reverse
22. I buy things on impulse.
25. I spend or charge more than I earn.
26. I often have extraneous thoughts when thinking.

BIS response scores:
Rarely/never (1)
Occasionally (2)
Often (3)
Almost always/Always (4)

Eating Attitudes Test (EAT)

1. I am terrified about being overweight.
11. I am preoccupied with a desire to be thinner.
12. I think about burning up calories when I exercise.
14. I am preoccupied with the thought of having fat on my body.

EAT response scores:
Always (3)
Usually (2)
Often (1)
Sometimes (0)
Rarely (0)
Never (0)

Obsessive Compulsive Inventory-Revised (OCI)

1. I have saved up so many things that they get in the way.
2. I check things more often than necessary.
4. I feel compelled to count while I am doing things.
6. I find it difficult to control my own thoughts.
7. I collect things I don't need.
9. I get upset if others change the way I have arranged things.
11. I sometimes have to wash or clean myself simply because I feel contaminated.
12. I am upset by unpleasant thoughts that come into my mind against my will.
13. I avoid throwing things away because I am afraid I might need them later.
16. I feel that there are good and bad numbers.
18. I frequently get nasty thoughts and have difficulty in getting rid of them.

OCI response scores:
Not at all (0)
A little (1)
Moderately (2)

A lot (3)
Extremely (4)

Zung Self-Rating Depression Scale (SDS)

11. My mind is as clear as it used to be. Reverse
12. I find it easy to do the things I used to. Reverse
13. I am restless and can't keep still.
14. I feel hopeful about the future. Reverse
16. I find it easy to make decisions. Reverse
17. I feel that I am useful and needed. Reverse
18. My life is pretty full. Reverse
20. I still enjoy the things I used to do. Reverse

SDS response score:
A little of the time (1)
Some of the time (2)
Good part of the time (3)
Most of the time (4)

State Trait Anxiety Inventory, Trait Subscale (STAI)

1. I feel pleasant. Reverse
3. I feel satisfied with myself. Reverse
5. I feel like a failure.
8. I feel that difficulties are piling up so that I cannot overcome them.
9. I worry too much over something that really doesn't matter.
10. I am happy. Reverse
12. I lack self-confidence.
13. I feel secure. Reverse
16. I am content. Reverse
19. I am a steady person. Reverse
20. I get in a state of tension or turmoil as I think over my recent concerns and interests.

STAI response items:
Almost never (1)
Sometimes (2)
Often (3)
Almost always (4)

SUPPLEMENTARY RESULTS

The Impact of Increasing Trial Number of Measurements of Metacognition: Binned Trials

When examining the first 20 burn-in trials, there was a significant decrease in mean confidence for binned trials (β=-0.05, SE=0.003, t=-17.53, *p*<0.001) (Figure S1A, trials 1-20). For the subsequent 80 main game trials, mean confidence increased for binned trials (β=0.006, SE=0.001, t=3.93, *p*<0.001) (Figure S1A, trials 21-100). Excluding the burn-in block rating, binned mean global evaluations increased from blocks 2 to 5 (β=0.04, SE=0.01, t=8.48, *p*<0.001) (Figure S1B). In contrast, accuracy significantly declined during for the binned burn-in trials (β=-0.03, SE=0.004, t=-8.94, *p*<0.001) and seemed to continue to decline for binned trials in the main game (β=-0.04, SE=0.002, t=-19.77, *p*<0.001) (Figure S1C), although the model failed to converge due to low variance in main game accuracy values. Mean dot difference (task difficulty level) declined during the burn-in trials (β=-0.27, SE=0.002, t=-118.9, *p*<0.001) and main game trials (β=-0.18, SE=0.001, t=-173.9, *p*<0.001) (Figure S1D).

Figure S1E shows the association between mean confidence with anxious-depression and compulsivity and intrusive thought across trial numbers, controlling for age, gender, levels of education in the model. The association between local confidence and anxious-depression remained stable from the 40th to the 100th trial, with no significant interaction effect of anxious-depression and trial number on mean confidence (β=0.001, SE=0.002, t=0.49, *p*=0.625) (Figure S1E). Similarly, the positive association between local confidence and compulsivity and intrusive remained stable from the 40th to 100th binned trial, with no significant interaction effect of trial number and compulsivity and intrusive thought on mean confidence (β=-0.002, SE=0.003, t=-0.68, *p*=0.494) (Figure S1E). Therefore, clinical correlates with local confidence were stable across the binned main game play trials. Excluding the burn-in block, there was no significant interaction effect of binned block number with anxious-depression (β=-0.003, SE=0.01, t=-0.33, *p*=0.742) or compulsivity and intrusive thought (β=0.01, SE=0.01, t=0.93, *p*=0.355) on mean global evaluations, indicating clinical correlates with global evaluations were stable across blocks (Figure S1F).

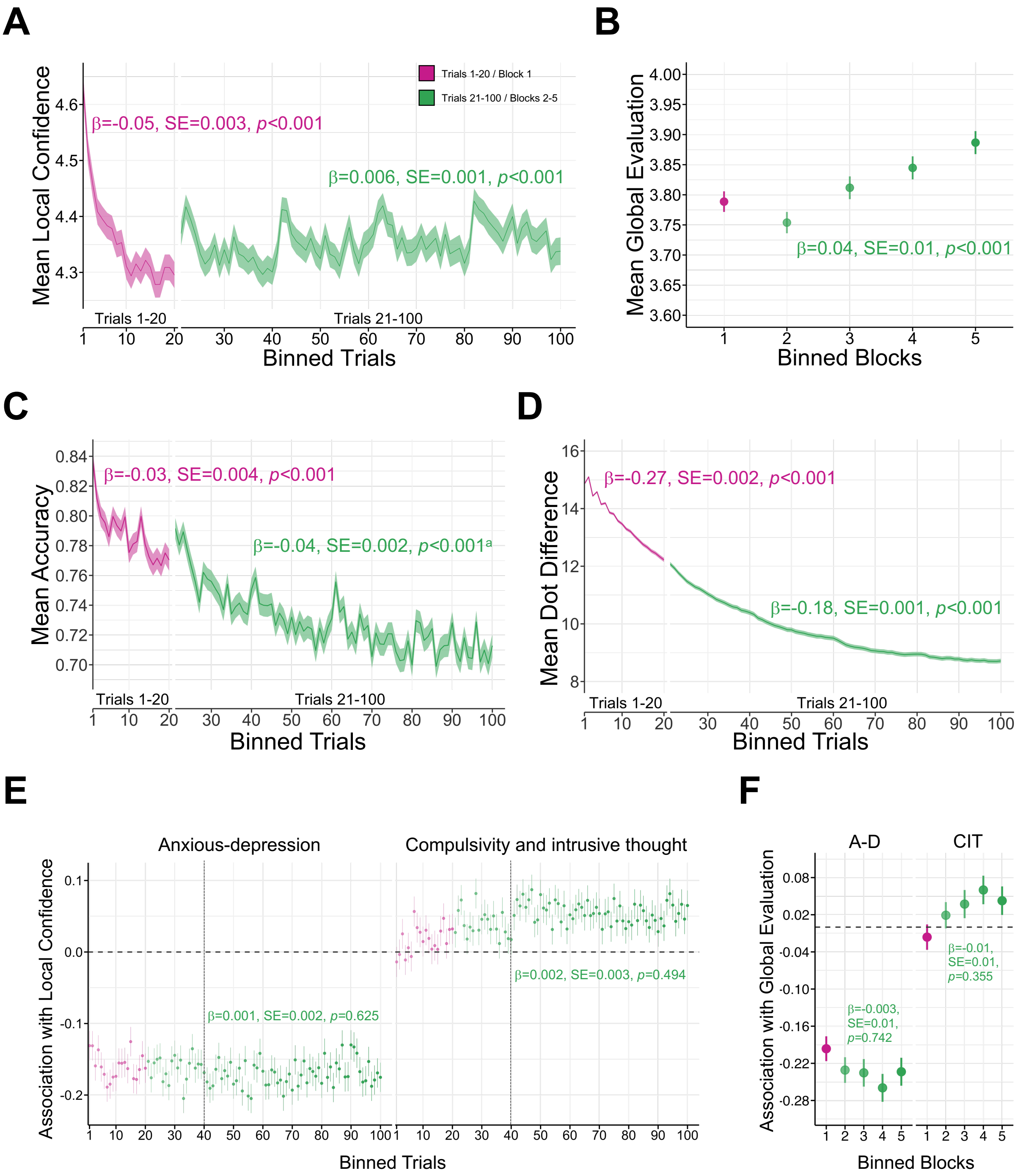

Fig. S1 The impact of increasing number of trials on mean local confidence, global evaluations, and accuracy with binned trial/block numbers (N=3388). β=standardised beta coefficient, *p*=p-value, [a]=mixed model is singular (did not converge), A-D = anxious-depression, CIT = compulsivity and intrusive thought The error bars represent the standard error around the standardised beta coefficient. **(A)** While mean confidence significantly reduced during the burn-in period for the staircase (pink line, trials 1-20), confidence estimates then increased across the remaining 80 main game trials (green line, trials 21-100). **(B)** Following the burn-in block (pink bar, block 1), mean global evaluations increased across blocks (green error bars, blocks 2-5). **(C)** Mean accuracy and **(D)** dot difference (task difficulty) declined for binned burn-in and then continued to decline for main game trials. **(E)** The significant associations between mean local confidence with anxious-depression and compulsivity and intrusive remained stable from the 40th (vertical dotted grey lines) to the 100th trial. **(F)** Following the burn-in block (pink error bar, block 1), there was no significant interaction effects of block number and transdiagnostic dimensions on mean global evaluation, indicating that associations with global evaluations remained stable across binned round-level ratings (green error bars, blocks 2-5).

SUPPLEMENTARY REFERENCES

1. Wise, T. & Dolan, R. J. Associations between aversive learning processes and transdiagnostic psychiatric symptoms in a general population sample. *Nat Commun* **11**, 4179 (2020).
2. Zung, W. W. A self-rating depression scale. *Arch Gen Psychiatry* **12**, 63–70 (1965).
3. Spielberger, C., Gorsuch, R., Lushene, R., Vagg, P. & Jacobs, G. *Manual for the State-Trait Anxiety Inventory (Form Y1 – Y2)*. Palo Alto, CA: Consulting Psychologists Press; vol. IV (1983).
4. Patton, J. H., Stanford, M. S. & Barratt, E. S. Factor structure of the Barratt impulsiveness scale. *J Clin Psychol* **51**, 768–774 (1995).
5. Foa, E. B. *et al.* The Obsessive-Compulsive Inventory: development and validation of a short version. *Psychol Assess* **14**, 485–496 (2002).
6. Garner, D. M., Olmsted, M. P., Bohr, Y. & Garfinkel, P. E. The eating attitudes test: psychometric features and clinical correlates. *Psychol Med* **12**, 871–878 (1982).
7. Marin, R. S., Biedrzycki, R. C. & Firinciogullari, S. Reliability and validity of the Apathy Evaluation Scale. *Psychiatry Res* **38**, 143–162 (1991).
